# Deep Learning Models for Evaluating the Anatomical Relationship Between Posterior Maxillary Teeth and Maxillary Sinus in Panoramic Radiographs

**DOI:** 10.1002/cre2.70264

**Published:** 2025-12-15

**Authors:** Akram Fallah, Parisa Soltani, Mojdeh Mehdizadeh, Mostafa Riahi Farsani, Mahsa Moannaei, Seyed Amir Hossein Ourang, Maryam Hossaini, Gianrico Spagnuolo, Carlo Rengo

**Affiliations:** ^1^ Department of Oral and Maxillofacial Radiology, Dental Implants Research Center, Dental Research Institute, School of Dentistry Isfahan University of Medical Sciences Isfahan Iran; ^2^ Department of Neurosciences, Reproductive and Odontostomatological Sciences University of Naples ‘Federico II’ Naples Italy; ^3^ Department of Chemistry, Faculty of Science Shahrekord University Shahrekord Iran; ^4^ Department of Oral and Maxillofacial Radiology of Hormozgan University Bandar Abbas Iran; ^5^ Dentofacial Deformities Research Center, Research Institute of Dental Sciences Shahid Beheshti University of Medical Sciences Tehran Iran

**Keywords:** cone beam computed tomography, convolutional neural networks, deep learning, maxillary sinus, panoramic radiography, posterior maxillary teeth

## Abstract

**Objectives:**

Accurate evaluation of the anatomical relationship between posterior maxillary teeth and the maxillary sinus is critical in dental treatments such as orthodontics, surgery, and implantology. This study investigates the efficacy of deep learning models applied to panoramic images for predicting this anatomical relationship.

**Materials and Methods:**

A total of 300 panoramic images and 1760 cropped slices were collected and converted to PNG format at a resolution of 512 × 512 pixels. Three convolutional neural network architectures—VGG, ResNet, and ResNeXt—were trained and evaluated. The dataset was split into training (80%), validation (10%), and testing (10%) sets. Performance metrics included accuracy, precision, recall, F1 score, ROC‐AUC, and confusion matrices.

**Results:**

The VGG model achieved an accuracy, precision, recall, and F1 score of 0.84, with an ROC‐AUC of 0.89. ResNet and ResNeXt demonstrated superior performance with all metrics at 0.88 and ROC‐AUC values of 0.93 and 0.94, respectively. Both ResNet and ResNeXt showed signs of overfitting after 30–50 epochs, suggesting the need for early stopping or regularization. False positives mainly occurred in second molars falsely classified as contacting the sinus.

**Conclusion:**

Deep learning models, particularly ResNet and ResNeXt, provide reliable assessment of the anatomical relationship between posterior maxillary teeth and the maxillary sinus using panoramic radiographs. These models can serve as efficient diagnostic aids when CBCT is unavailable, improving clinical decision‐making in panoramic images.

## 1 Introduction

The maxillary sinus, recognized as the largest paranasal sinus, serves multiple functions, including contributing to voice resonance, shaping the facial structure, regulating inhaled air temperature and humidity, and acting as a shock absorber for impacts to the facial area (Alqahtani et al. 2020; Som and Curtin 2020). Anatomically, this air‐filled cavity is located adjacent to the apices of the posterior maxillary teeth (Ding et al. 2024; Sarilita et al. 2024), a proximity that can predispose to complications such as odontogenic sinusitis caused by periodontal disorders, periapical infections, or trauma resulting from therapeutic interventions (Oscar et al. 2010).

Approximately 10%–12% of maxillary sinusitis cases have an odontogenic origin, and if left untreated, these infections may extend to surrounding structures, including the orbital cavity and even the cranial vault (Little et al. 2018). Procedures such as tooth extraction or endodontic surgery may result in perforation of the sinus floor, creation of an oroantral fistula, or displacement of root fragments into the maxillary sinus (Harrison 2018). Therefore, accurate assessment of the anatomical relationship between the root apices of the posterior maxillary teeth and the sinus floor is of significant importance in diagnosis and treatment planning for the posterior maxilla.

Currently, cone‐beam computed tomography (CBCT) is considered the gold standard for three‐dimensional evaluation of the tooth–sinus relationship (Regnstrand et al. 2021). However, its use is limited by high costs, relatively high radiation doses, and time‐consuming data interpretation (Hawon and Andreu 2021; Marcu et al. 2018). In contrast, panoramic radiography is widely used due to its simplicity, low radiation dose, low cost, and broad coverage of the maxillofacial region (Shrestha et al. 2022). Nevertheless, overlapping structures, dimensional distortion, and lack of cross‐sectional information can lead to misinterpretation of the sinus position relative to posterior teeth (Shrestha et al. 2022).

Artificial intelligence (AI), defined as the ability of machines to perform complex tasks by mimicking human cognitive processes, has shown remarkable progress in recent years, particularly in radiologic image interpretation (Orhan et al. 2021). Among AI approaches, deep learning systems based on convolutional neural networks (CNNs) have gained special attention (Soltani et al. 2024). The use of AI models in the analysis of maxillofacial radiographic images—including CBCT, panoramic, and periapical radiographs—is increasing. In panoramic image interpretation, various models have been employed, such as CNNs, dense neural networks, computer vision algorithms, and hybrid approaches.

CNNs, which process data using convolutional filters, are among the most effective models for radiologic image analysis (Smith et al. 2023). In dentistry, AI has been applied to detect dental caries, periapical lesions, periodontal disease, and estimate dental age (Lee et al. 2022). U‐Net and Mask R‐CNN models have proven effective in precise segmentation of teeth and the maxillary sinus (Murphy 2012). Furthermore, combining CNNs with other models and using image preprocessing techniques can enhance diagnostic accuracy (Russell and Norvig 2020).

Although extensive research has been conducted on interpreting maxillofacial and sinus radiographs, studies investigating the anatomical relationship between the maxillary sinus floor and posterior teeth using AI remain limited. The only notable study in this field is by Ding et al. (2024) in China, who employed U‐Net and DenseNet models to analyze 1,035 panoramic images, achieving an accuracy of 0.89 and sensitivity of 0.85—outperforming both dentists and radiologists.

Given the complexity of panoramic image interpretation and the limitations of available data, the present study aims to utilize three AI models and employ CBCT as the gold standard to identify the three‐dimensional anatomical relationship between posterior teeth and the maxillary sinus. The ultimate goal is to develop an intelligent tool to assist clinicians in improving diagnostic accuracy, reducing interpretation time, and lowering costs. The results of this study could contribute to the development of an effective AI algorithm for precise analysis of the anatomical relationship between posterior maxillary teeth and the maxillary sinus in panoramic radiographs.

## 2 Materials and Methods

### 2.1 Ethical Approval

The study protocol was approved by the Ethics Committee of Isfahan University of Medical Sciences (Approval No.: IR. MUI. DHMT. REC.1404.054). The report follows the “Artificial Intelligence in Dental Research” checklist.

### 2.2 Study Design and Data Collection

This cross‐sectional study used 300 panoramic radiographs and corresponding CBCT scans from the archives of the Department of Oral and Maxillofacial Radiology, Isfahan University of Medical Sciences, Iran, and two additional centers between December 2024 and April 2025. Images were obtained from patients over 18 years old for diagnostic reasons other than the purpose of this study, with all personal identifiers removed in compliance with ethical standards.

Panoramic radiographs were acquired using a Planmeca Plus system (Planmeca, Helsinki, Finland), and CBCT scans of the same patients were obtained using a Dentsply Sirona Orthophos SL 3D scanner (Dentsply Sirona, Bensheim, Germany). Exposure parameters were adjusted according to individual patient characteristics to optimize image quality, and in any case we have to provide a range of the parameters.

#### 2.2.1 Inclusion Criteria

1. Presence of posterior maxillary teeth without periapical lesions (only the tooth presenting with a lesion is excluded from the study, while the remaining teeth of the patient are included for evaluation).
2. No history of dental diseases affecting the maxillary sinus.
3. Absence of traumatic injuries altering the normal sinus anatomy.
4. Availability of CBCT scans of the posterior teeth taken at most 3 months after the panoramic radiograph.

#### 2.2.2 Exclusion Criteria

- Low‐quality panoramic or CBCT images.
- History of special treatments involving the maxillofacial region.

A total of 300 panoramic images were collected. To improve algorithm performance in identifying teeth and the maxillary sinus, images were cropped into smaller regions containing the premolar and molar areas on both right and left sides, yielding 1760 cropped images. Of these, 931 were classified as “contact” and 829 as “no contact.” Two experienced oral and maxillofacial radiologists independently assessed the relationship between posterior maxillary teeth and the maxillary sinus on CBCT images (in both cross‐sectional and axial views). Based on these findings, corresponding panoramic images were labeled as “contact” or “no contact” (Regnstrand et al. 2021).

### 2.3 Data Preparation

All images were anonymized and coded before analysis. The dataset was split into training (80%), validation (10%), and test (10%) sets. The training set contained 1584 cropped images (843 contact, 741 no contact), the validation set included 176 images (88 contact, 88 no contact), and the test set comprised 176 images (88 contact, 88 no contact) not used in training. To ensure that the artificial intelligence model can perform effectively in various dental conditions—including restorations, crowns, decay, or the presence of endodontic materials inside the canals—the model was trained using all teeth exhibiting the aforementioned conditions, as well as completely healthy teeth without any decay.

### 2.4 Data Labeling

**Figure 1:**
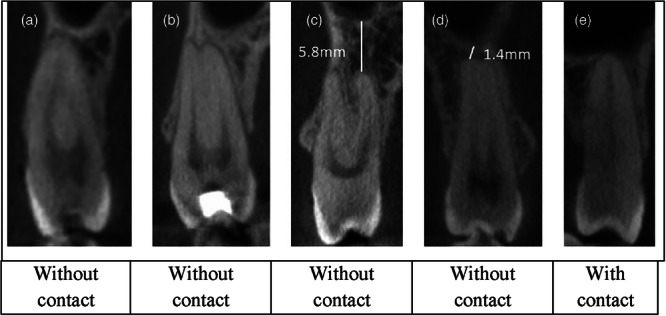
An example of cone‐beam computed tomography images for evaluating the contact between teeth and the sinus (a–d) without contact and (e) with contact.

**Figure 2:**
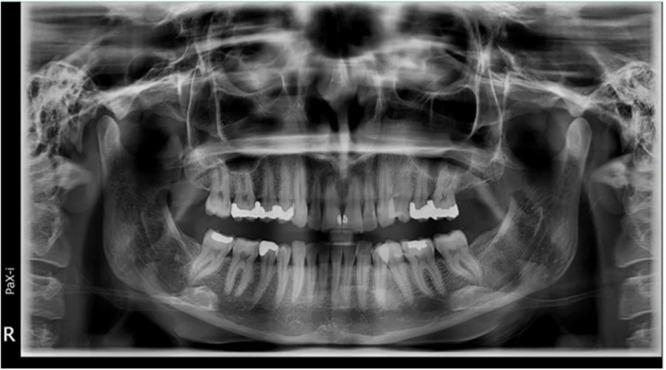
Representative panoramic image.

**Figure 3:**
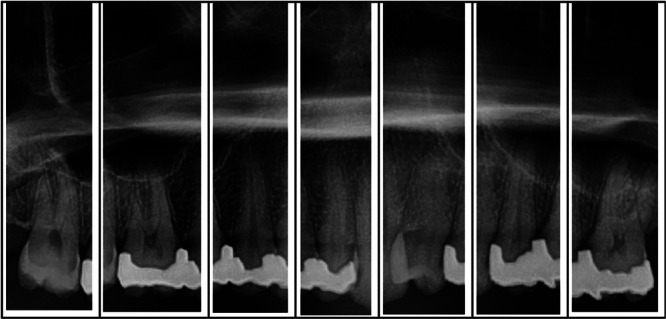
Sectional view of the posterior maxillary teeth in panoramic radiography.

In cases of disagreement, both observers reviewed the images together until consensus was reached (Figures 1, 2, 3).

### 2.5 Region of Interest (ROI) Selection

On panoramic images, each tooth under study was cropped based on anatomical boundaries:

- Lower boundary: 2 mm below the occlusal surface of the tooth.
- Upper boundary: Extending into the maxillary sinus.
- Anterior boundary: Distal half of the preceding tooth.
- Posterior boundary: Mesial half of the following tooth.

### 2.6 Image Preprocessing

Panoramic radiographs and CBCT scans were collected in DICOM format. Before analysis, all images were converted to PNG format to facilitate processing. Subsequently, each image was resized to a standard resolution of 512 × 512 pixels to ensure uniformity across the dataset. To artificially expand the training dataset and improve model robustness, data augmentation techniques were applied, including random rotations of up to ±10°, horizontal flipping, and brightness adjustment.

### 2.7 Models and Model Parameters

Three deep learning models—VGG, ResNet, and ResNeXt—were implemented to assess the presence or absence of contact between posterior maxillary teeth and the maxillary sinus. A total of 1584 cropped images from the dataset (843 contact, 741 no contact) were used for initial model training. An additional 176 images served as the validation set to monitor performance during training. The final evaluation was conducted using 176 independent test images, equally divided between the two categories, which were not included in the training phase.

All models were based on convolutional neural networks (CNNs) comprising an input layer, an output layer, and multiple hidden layers. Each layer consisted of interconnected computational units (“nodes” or “neurons”) that process visual information. Upon input, each image was divided into patches and fed to the network's input layer. The data then passed sequentially through the hidden layers, where each node applied specific mathematical operations to modify and transmit values to subsequent nodes. This process continued until the output layer, where aggregated information from all nodes generated the final classification result.

### 2.8 Model Performance Evaluation

To assess model performance in detecting anatomical structures and classifying the tooth–sinus relationship, the following metrics were calculated: accuracy, precision, error rate, recall (sensitivity), F1 score, confusion matrix, and the area under the receiver operating characteristic curve (AUC–ROC). The formulas for each metric, as adapted from the original text, are as follows:

- **Accuracy**—a general measure of how often the model correctly predicts the output:
$$\mathrm{Accuracy}=\frac{\mathrm{TP}+\mathrm{TN}}{\mathrm{TP}+\mathrm{FN}+\mathrm{FP}+\mathrm{TN}}$$
  where *TP* = true positives, *TN* = true negatives, *FP* = false positives, and *FN* = false negatives.
- **Precision**—measures the proportion of cases predicted as positive that are actually positive:
$$\mathrm{Precision}=\frac{\mathrm{TP}}{\mathrm{TP}+\mathrm{FN}}$$
- **Recall (sensitivity)**—measures the proportion of actual positive cases correctly identified by the model:
$$\mathrm{Recall}=\frac{\mathrm{TP}}{\mathrm{TP}+\mathrm{FP}}$$
- **Error rate**—the proportion of incorrect predictions relative to the total predictions, and the complement of accuracy:
$$\mathrm{Error}=\frac{\mathrm{FP}+\mathrm{FN}}{\mathrm{TP}+\mathrm{FN}+\mathrm{FP}+\mathrm{TN}}$$
Error=1−Accuracy
- **F1 score**—the harmonic mean of precision and recall:
$$F1=2\times \frac{\mathrm{Precision}\times \mathrm{Recall}}{\mathrm{Precision}+\mathrm{Recall}}$$
- **Confusion matrix**—summarizes classification results by categorizing outcomes into four classes: true positives (TP), true negatives (TN), false positives (FP), and false negatives (FN). From these values, accuracy, recall, precision, and F1 score can be derived.

These metrics were comprehensively applied to evaluate model performance in classifying the anatomical relationship between posterior maxillary teeth and the maxillary sinus.

## 3 Results

A total of 300 panoramic radiographs were collected, yielding 1760 cropped images for analysis: 931 in the “contact” group and 829 in the “no contact” group. Data were split in an 80:10:10 ratio for training, validation, and testing, respectively. The training set contained 1584 images (843 contact, 741 no contact), the validation set 176 images (88 contact, 88 no contact), and the test set 176 images (88 contact, 88 no contact). The test set was not used during training. Three convolutional neural network (CNN) models—VGG, ResNet, and ResNeXt—were evaluated for their ability to classify the anatomical relationship between posterior maxillary teeth and the maxillary sinus.

### 3.1 Tooth–Sinus Contact Classification

#### 3.1.1 Vgg Model

**Figure 4:**
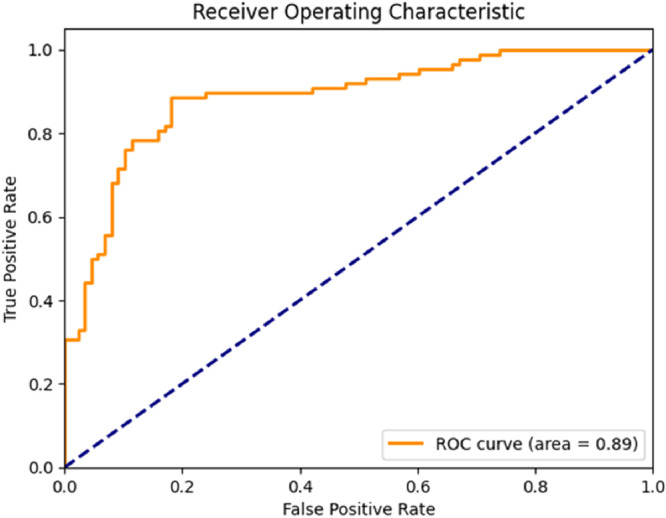
ROC curve of the VGG model.

**Figure 5:**
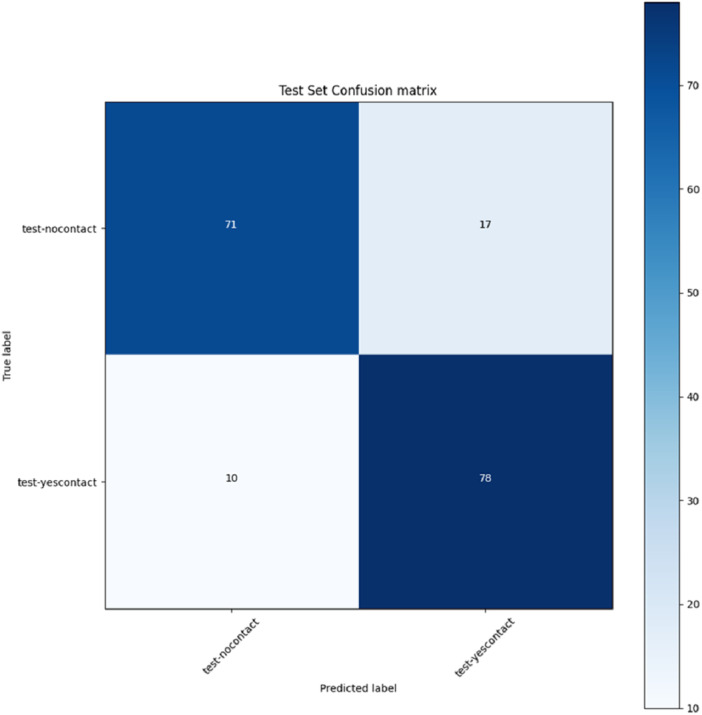
Confusion matrix of the VGG model.

Following training, the VGG model achieved an accuracy of 0.84, precision of 0.84, recall of 0.84, and F1 score of 0.84 on the test set. The area under the ROC curve (AUC) was 0.89 (Figure 4), indicating good discriminative performance (AUC range 0.80–0.90). The confusion matrix (Figure 5) showed that, of 88 contact cases in the test set, 78 were correctly classified (true positives) and 10 were misclassified (false negatives). Of 88 no‐contact cases, 71 were correctly classified (true negatives), and 17 were misclassified (false positives).

#### 3.1.2 Resnet Model

**Figure 6:**
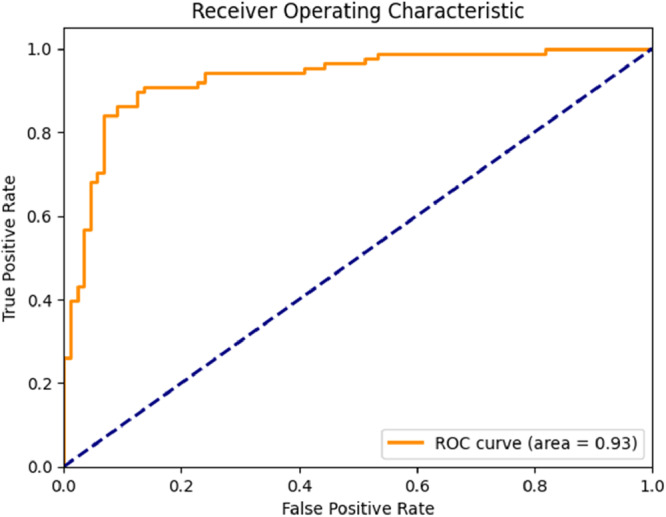
ROC curve of the ResNet model.

**Figure 7:**
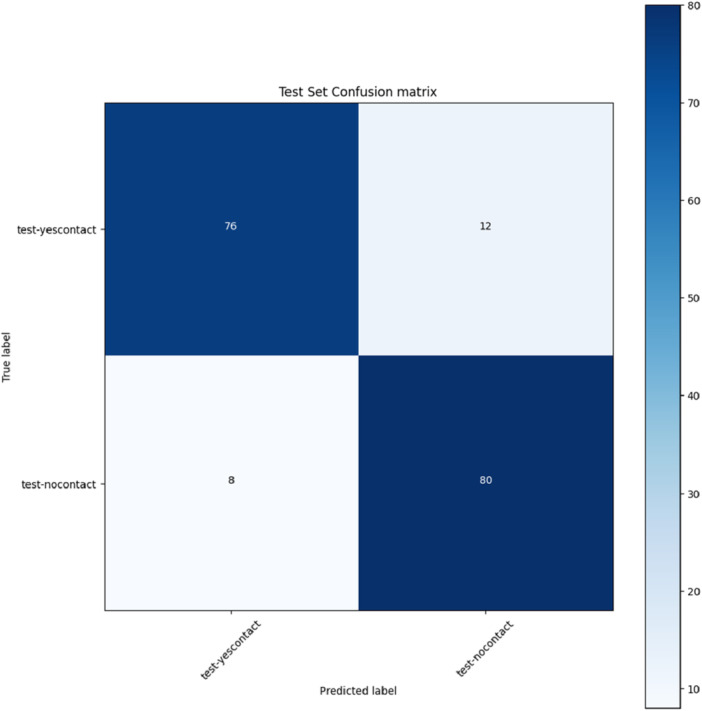
Confusion matrix of the ResNet model.

The ResNet model achieved an accuracy of 0.88, precision of 0.88, recall of 0.88, and F1 score of 0.88. The AUC was 0.93 (Figure 6), indicating excellent performance (AUC > 0.90). In the confusion matrix (Figure 7), the model correctly classified 76 contact cases and misclassified 12, while correctly classifying 80 no‐contact cases and misclassifying 8.

**Figure 8:**
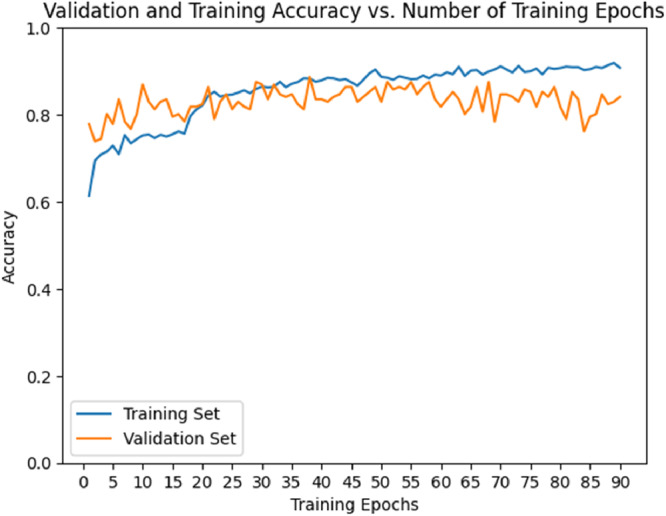
Validation and training accuracy of the ResNet model.

**Figure 9:**
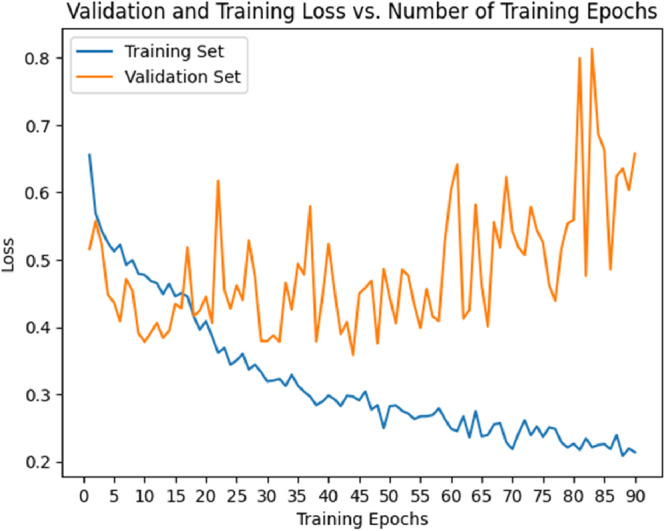
Validation and training loss in the ResNet model.

The accuracy curve (Figure 8) demonstrated a steady increase in training accuracy, reaching approximately 0.80–0.90 by epoch 50 and then plateauing. Validation accuracy also increased but remained lower (0.60–0.70) and showed greater fluctuations after 50 epochs. The loss curve (Figure 9) showed a decrease in training loss, stabilizing near 0.20, while validation loss initially decreased but began to rise and fluctuate (~0.80) after 50 epochs, suggesting overfitting. Thus, ResNet exhibited strong learning initially but signs of overfitting emerged after ~50 epochs.

#### 3.1.3 Resnext Model

**Figure 10:**
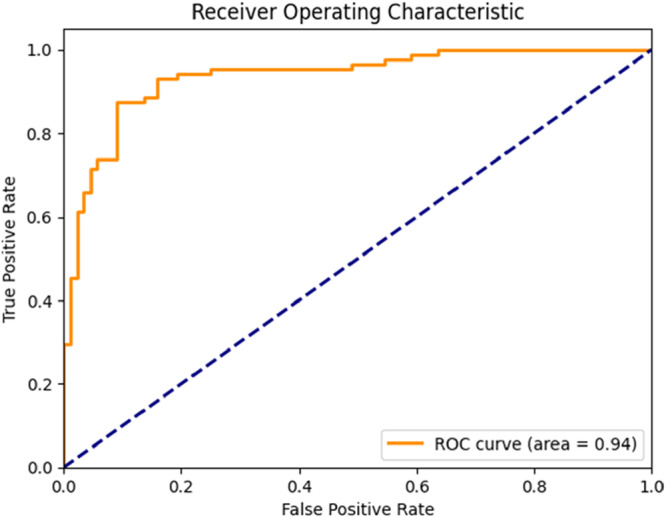
ROC curve of the ResNeXt model.

**Figure 11:**
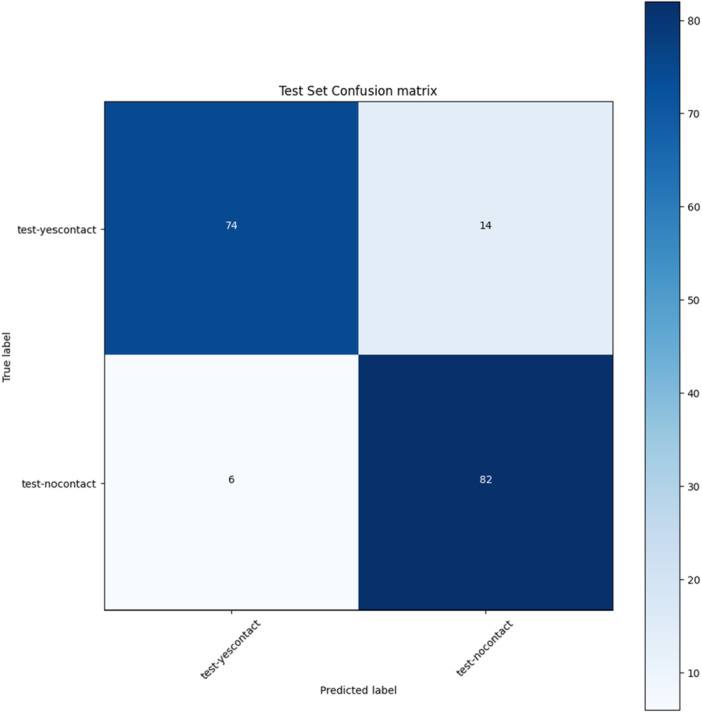
Confusion matrix of the ResNeXt model.

The ResNeXt model achieved an accuracy of 0.88, precision of 0.88, recall of 0.88, and F1 score of 0.88. The AUC was 0.94 (Figure 10), also indicating excellent performance. The confusion matrix (Figure 11) showed that 74 contact cases were correctly classified and 14 misclassified, while 82 no‐contact cases were correctly classified and 6 misclassified.

**Figure 12:**
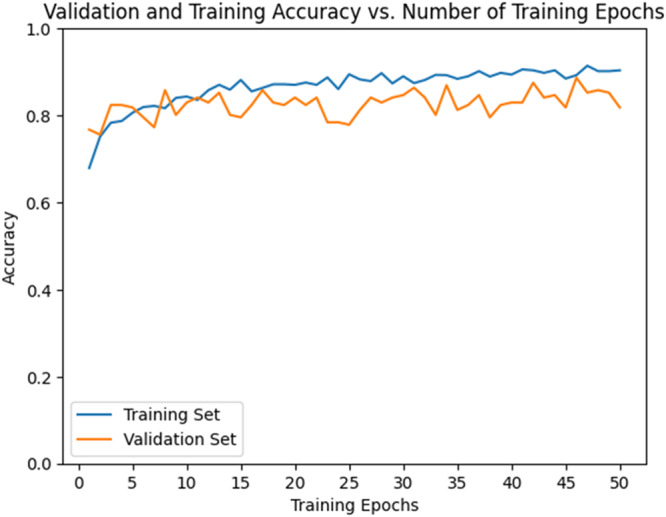
Validation and training accuracy of the ResNeXt model.

**Figure 13:**
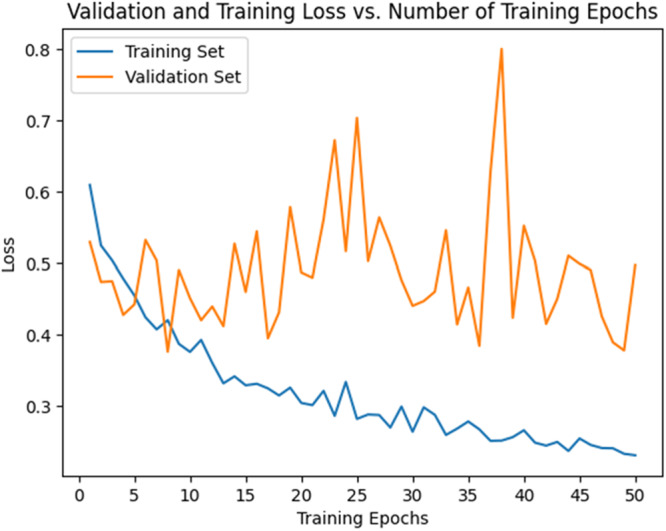
Validation and training loss in the ResNeXt model.

The accuracy curve (Figure 12) showed training accuracy increasing steadily, reaching 0.80–0.90 by epoch 30 and plateauing thereafter. Validation accuracy also increased but stabilized at ~0.60–0.70 and remained lower than training accuracy. The loss curve (Figure 13) showed training loss decreasing to ~0.30–0.40 by epochs 30–40, while validation loss decreased initially but began fluctuating and increasing after epoch 30—again indicating overfitting. Based on these findings, it is recommended to stop training ResNeXt at 30–40 epochs or apply techniques such as dropout, regularization, or early stopping to improve generalization.

### 3.2 Summary of Model Performance

All three models performed well on the training data, with ResNet and ResNeXt achieving higher AUC values than VGG. However, both ResNet and ResNeXt showed signs of overfitting, indicating the need for regularization strategies. Detailed results for all models are summarized in Table 1.

**Table 1:** Performance results of the artificial intelligence models.

AI models	VGG	ResNet	ResNeXt
Accuracy	0.84	0.88	0.88
Precision	0.84	0.88	0.88
Recall	0.84	0.88	0.88
F1 score	0.84	0.88	0.88
AUC	0.89	0.93	0.94
Loss	0.50	0.37	0.40

## 4 Discussion

During various dental treatments, including orthodontics, surgery, endodontics, periodontology, and implant placement in the posterior maxilla, clinicians are faced with the challenge of the maxillary sinus in this area (Ding et al. 2024; Shrestha et al. 2022). The close relationship between the posterior maxillary teeth and the maxillary sinus can lead to various complications during treatment.

Given the potential complications arising from dental problems and dental treatments due to the proximity of posterior maxillary teeth to the maxillary sinus, accurate evaluation of this anatomical relationship is of great importance for diagnosis and treatment planning in the posterior maxilla.

Panoramic radiography is widely used for initial assessment of the relationship between the maxillary sinus and posterior teeth. When the sinus floor is clearly visible and separated from the posterior teeth in panoramic images, further evaluation with CBCT is unnecessary. However, when the sinus is close to the teeth, the limitations of panoramic imaging—such as superimposition of anatomical structures, distortion in vertical and horizontal dimensions, absence of cross‐sectional information, patient positioning errors, and ghost images—may result in a false depiction of the relationship between the sinus and posterior teeth (Yoo et al. 2021). In such cases, CBCT, which is considered the gold standard for assessing this anatomical relationship, is necessary. Nevertheless, access to CBCT is limited due to its higher cost and radiation dose compared to panoramic radiography, and the loading, analysis, and interpretation of CBCT data can be time‐consuming (Regnstrand et al. 2021; Hawon and Andreu 2021).

With the increasing application of artificial intelligence (AI) models in dentistry and radiology, it is now possible to use this technological advancement to interpret conventional 2D images such as panoramic and periapical radiographs. Deep learning plays a crucial role in diagnosing various conditions, including dental caries, jaw diseases, and fractures. The use of different algorithms can enhance the diagnostic potential of 2D images, and in cases where dentists have limited access to 3D CBCT images, AI can assist in improving diagnostic accuracy.

Given the diagnostic importance of the anatomical relationship between posterior maxillary teeth and the maxillary sinus, and the lack of sufficient studies in this field, we conducted a study to evaluate the performance of several AI models in detecting this relationship and compared the results with Ding's study (Ding et al. 2024).

In this research, VGG, ResNet, and ResNeXt were used for the detection of the anatomical relationship between posterior maxillary teeth and the maxillary sinus. VGG is a family of CNN models developed by the Visual Geometry Group at the University of Oxford, known for its simple and regular structure, consisting of convolutional layers with small 3×3 filters and max‐pooling layers (Simonyan and Zisserman 2015). ResNet is a network architecture designed to address the vanishing gradient problem in deeper networks. It introduces residual or shortcut connections that pass the output of one layer directly to subsequent layers, allowing easy transfer of information from earlier to deeper layers. This architecture enables networks to be deeper and achieve better performance in various computer vision tasks (He et al. 2016). ResNeXt is an enhanced version of ResNet that incorporates the concept of residual blocks with greater diversity. Specifically, ResNeXt uses multiple parallel paths with identical structure within each residual block, enabling the network to learn more complex features with fewer parameters than wider or deeper networks, striking a good balance between accuracy and computational efficiency (Xie et al. 2017).

Our results showed that the VGG model achieved an accuracy of 0.84, sensitivity of 0.84, and an F1 score of 0.84. Compared to the DenseNet model used in Ding's study, VGG demonstrated higher accuracy and similar sensitivity, indicating acceptable performance. For ResNet, the accuracy, sensitivity, and F1 score were each 0.88, higher than those reported for DenseNet in Ding's study, indicating superior performance. Similarly, the ResNeXt model achieved an accuracy, sensitivity, and F1 score of 0.88, also outperforming DenseNet in Ding's study.

When comparing the three AI models used in the present study, ResNeXt and ResNet both demonstrated higher accuracy, sensitivity, and F1 scores compared to VGG. Despite ResNeXt being a newer generation of ResNet, their performance was essentially the same in our evaluation. Overall, all three models demonstrated reliable performance for predicting the anatomical relationship between posterior maxillary teeth and the maxillary sinus. Since these models were trained using CBCT, the diagnostic gold standard, they represent highly suitable tools for this important clinical application.

Analysis of the test dataset for all three models revealed that the most common error occurred when the second molar, which had no contact with the sinus, was misclassified as having contact. This false positive finding may indicate that in the posterior maxilla, as we move further posteriorly, the likelihood of superimposition of anatomical structures increases. Nevertheless, given the low frequency of such errors relative to the overall strong performance of these models, this issue can be considered clinically acceptable.

This study has several limitations. First, the sample size was relatively small, as it was challenging to identify pairs of panoramic and CBCT images that met the inclusion criteria. Second, all radiographic data were obtained from a single set of devices (Planmeca, panoramic scanner and CBCT scans using a Dentsply Sirona Orthophos SL 3D scanner), which may restrict the generalizability of the results. In addition, data collection for model training was time‐consuming, since both panoramic and CBCT images had to be available for each patient within the archives of participating centers. In practice, only a limited number of patients had both imaging modalities recorded at the same facility.

## 5 Conclusion

The VGG, ResNet, and ResNeXt models demonstrated acceptable levels of accuracy, sensitivity, and F1 score. Therefore, the use of these models is recommended for evaluating the anatomical relationship between posterior maxillary teeth and the maxillary sinus in panoramic images.

## Author Contributions

Akram Fallah, Parisa Soltani, and Mojdeh Mehdizadeh contributed equally to the conceptualization and design of the study. Akram Fallah, Mahsa Moannaei, and Seyed Amir Hossein Ourang were responsible for data collection and preprocessing of the panoramic radiographs. Mostafa Riahi Farsani and Seyed Amir Hossein Ourang performed the data analysis and deep learning model implementation. Mostafa Riahi Farsani and Maryam Hossaini contributed to the interpretation of results and drafting the manuscript. Gianrico Spagnuolo and Carlo Rengo provided supervision, critical revision, and final approval of the manuscript.

## Funding

The authors received no specific funding for this work.

## Ethics Statement

The authors have nothing to report.

## Conflicts of Interest

The authors declare no conflicts of interest.

## Data Availability

The data that support the findings of this study are available from thecorresponding author upon reasonable request.
